# Naturally Derived Heme-Oxygenase 1 Inducers and Their Therapeutic Application to Immune-Mediated Diseases

**DOI:** 10.3389/fimmu.2020.01467

**Published:** 2020-07-23

**Authors:** Samanta C. Funes, Mariana Rios, Ayleen Fernández-Fierro, Camila Covián, Susan M. Bueno, Claudia A. Riedel, Juan Pablo Mackern-Oberti, Alexis M. Kalergis

**Affiliations:** ^1^Departamento de Genética Molecular y Microbiología, Millenium Institute on Immunology and Immunotherapy, Facultad de Ciencias Biológicas, Pontificia Universidad Católica de Chile, Santiago, Chile; ^2^Departamento de Ciencias Biológicas, Millenium Institute on Immunolgy and Immunotherapy, Facultad Ciencias de la Vida, Universidad Andrés Bello, Santiago, Chile; ^3^Instituto de Medicina y Biología Experimental de Cuyo, IMBECU CCT Mendoza- CONICET, Mendoza, Argentina; ^4^Facultad de Ciencias Médicas, Instituto de Fisiología, Universidad Nacional de Cuyo, Mendoza, Argentina; ^5^Departamento de Endocrinología, Facultad de Medicina, Pontificia Universidad Católica de Chile, Santiago, Chile

**Keywords:** heme oxygenase 1, HO-1, autoimmunity, naturally derived compounds, inflammation

## Abstract

Heme oxygenase (HO) is the primary antioxidant enzyme involved in heme group degradation. A variety of stimuli triggers the expression of the inducible HO-1 isoform, which is modulated by its substrate and cellular stressors. A major anti-inflammatory role has been assigned to the HO-1 activity. Therefore, in recent years HO-1 induction has been employed as an approach to treating several disorders displaying some immune alterations components, such as exacerbated inflammation or self-reactivity. Many natural compounds have shown to be effective inductors of HO-1 without cytotoxic effects; among them, most are chemicals present in plants used as food, flavoring, and medicine. Here we discuss some naturally derived compounds involved in HO-1 induction, their impact in the immune response modulation, and the beneficial effect in diverse autoimmune disorders. We conclude that the use of some compounds from natural sources able to induce HO-1 is an attractive lifestyle toward promoting human health. This review opens a new outlook on the investigation of naturally derived HO-1 inducers, mainly concerning autoimmunity.

## Introduction

Heme oxygenase (HO, EC 1.14.99.3) is a microsomal enzyme first described in 1968 (1) with a primary antioxidant and anti-inflammatory role involved in heme group degradation yielding carbon monoxide (CO), biliverdin, and free iron (2). To date, three HO isoenzymes (HO-1, HO-2, and HO-3) have been reported in mammals. Of these three isoenzymes, only HO-1 has been demonstrated to be inducible in response to a variety of stimuli (3, 4). The beneficial effect of HO-1 induction in inflammation has been associated not only with the degradation of the heme group but also with its anti-inflammatory products, biliverdin, and CO (5, 6).

Importantly, HO-1 induction is triggered by its substrate heme and by biological, chemical, and physiological stress conditions caused by toxic concentrations of drugs or metals (7). Therefore, HO-1 induction is actively involved in the oxidative response, and its induction has been used as an approach for the treatment of inflammatory diseases (8–11).

Many natural compounds have shown an effective induction of HO-1 without cytotoxic effects. Most of them are chemicals present in plants used as food, spices, flavoring, and medicine (7). In this review article, we will discuss some naturally derived compounds known to up-regulate the expression of HO-1, the molecular mechanisms involved in HO-1 induction, and the beneficial effects of these natural compounds in different autoimmune disorders.

## Molecular Mechanism of HO-1 Induction

Heme oxygenase 1 has great therapeutic potential value given that in several conditions and diseases, there are stress factors that induce the expression of HO-1 activity, reducing the inflammation. Therefore, it seems essential to better know the molecular mechanisms involved in the induction of the HO-1 expression and the regulation of its activity. In this section, we discuss the role of transcription factors and upstream signaling molecules in the modulation of HO-1 expression.

### Transcription Factors

The HO-1 gene (*hmox1*) is often activated under a wide range of stressful conditions. The transcriptional control of *hmox1* is determined by inducible regulatory elements localized in the 5′ region of the promoter (4, 12). Distal enhancers regions identifying upstream *hmox1* (13, 14) are critical in HO-1 induction by different stimuli and contain several stress-responsive elements with binding sites for regulatory proteins (15, 16). Several redox-sensitive transcription factors bind to these elements, and some of them will be discussed below.

#### Nuclear Factor–Erythroid 2–Related Factor 2

Nuclear factor–erythroid 2–related factor 2 (Nrf2) is a transcription factor that regulates the expression of proteins functionally related to detoxification, reduction of oxidized proteins, and the elimination of end products derived from reactive oxygen species (ROS) (17). Nuclear factor–erythroid 2–related factor 2 binds to small Maf protein forming a heterodimer, and then this dimer can bind to the antioxidant response element (ARE) or Maf recognition elements (MAREs) (18). These sequences are present at the HO-1 promoter; thus, the Nrf2-Maf dimer binding induces the transcriptional expression of HO-1 mRNA (19).

The activity of Nrf2 is normally repressed by the repressor Kelch-like ECH-associated protein 1 (Keap1), which sequestrate Nrf2 in the cytoplasm (20). Some electrophilic agents and ROS alter the interaction of Nrf2-Keap1 and liberate Nrf2 activity from repression (21). Moreover, Bach1, another transcriptional repressor, competes for binding, and forming heterodimers with small Maf proteins. These dimers bind to MAREs at the DNA repressing HO-1 transcription (22). However, an inductor such as heme binds to four cysteine–proline motifs in the C-terminal region of Bach and inhibits the DNA-binding activity of Bach1–Maf heterodimers resulting in HO-1 induction (23).

#### Activator Protein 1

The activator protein 1 (AP-1) transcription factor is a dimer of Jun and Fos family proteins (24). Activator protein 1 is involved in the induction of immune responses in a great diversity of ways, including different tissues and immune or non-immune cell types (25, 26). Interestingly enough, AP-1 homodimers or heterodimers bind to enhancers flanking the promoter region of *hmox1* (14). The induction of HO-1 expression requires AP-1 activation to respond to some oxidative inducers (27, 28), such as lipopolysaccharide (LPS) (29).

### Upstream Signaling Molecules

The activation of the transcription factors mentioned above can be indirectly modulated by various proteins with (de)phosphorylation or reduction–oxidation activity, such as the mitogen-activated protein kinases (MAPKs), phosphatidylinositol 3-kinase (PI3K), and other protein kinases, leading to HO-1 regulation. These signaling pathways and their association to the immune response will be described below.

#### Mitogen-Activated Protein Kinases

The activation of MAPKs has been suggested to play a critical role in HO-1 up-regulation (4). Among them, three major subfamilies of MAPKs have been described in HO-1 expression modulation: the extracellular regulated kinases (ERK), c-Jun N-terminal kinases (JNK), or stress-activated kinases, and p38 (4, 30). Thus, p38 function has been involved in the HO-1 induction by isoproterenol (31), ethanol extract of *Inula helenium* (32), and tetrahydroisoquinoline alkaloid THI-28 (33) in RAW 264.7 macrophages. In addition, khayandirobilide A, an anti-inflammatory compound from *Khaya senegalensis*, induces HO-1 expression by p38 MAPK/Nrf2 signaling in RAW 264.7 macrophages and BV-2 microglial cells (34). Besides, p38 inhibition has been reported to induce HO-1 expression mediated by Nrf2 in monocytes, (35) human leukocytes (36), and RAW 264.7 macrophages (37).

On the other hand, the induction of HO-1 expression by cadmium has been reported to be JNK and ERK pathway-mediated in the lymphocyte B-cell line BJAB cells in a dose-dependent manner (38). Moreover, inhibition of the JNK pathway is involved in the anti-inflammatory effects of kalopanaxsaponin A from *Kalopanax pictus* (39) and sulforaphane (SFN) (40) in LPS-stimulated microglia. Also, ethanol-treated rat Kupffer cells display increased mRNA expression of HO-1 mediated by Nrf2, hypoxia-inducible factor 1α, and JNK-1 (41). Moreover, HO-1 is induced in microglia by the activation of Nrf2 via the ERK signaling pathway under astragaloside IV (42) and artesunate treatment (43). Accordingly, the MAPK pathway involved HO-1 up-regulation, which probably is dependent on the cell type and inducer. Hence, more studies should be performed to improve the understanding of the mechanisms underlying the regulation of HO-1 expression.

#### Phosphatidylinositol 3-Kinase

In diverse models, it has been shown that HO-1 expression could be up-regulated via PI3K/Akt pathway and Nfr2 (44, 45). Most of the studies report the induction of HO-1–mediated via PI3K in immune cells described in cells from the innate system. Heme oxygenase 1 induction has also been reported to be PI3k mediated in RAW 264.7 macrophages after isoproterenol treatment (31). The anti-inflammatory effects of schisandrin from *Schisandra chinensis* in LPS-stimulated RAW 264.7 have been due to the induction of HO-1 expression through Nrf-2 and PI3K/Akt activation. Interestingly, the down-regulation of the PI3K/Akt signaling pathway increases Nrf2/HO-1 and inhibits mast cell degranulation (46). Besides, edaravone (a radical scavenging agent) reduces experimental autoimmune thyroiditis severity in a PI3K/Akt pathway-dependent way by inducing HO-1 (47).

#### Others Protein Kinases

Heme oxygenase 1 expression can involve different upstream signaling according to the cell type evaluated, as was mentioned before. Thus, several signaling cascades have been associated with HO-1 up-regulation, including protein kinase A (PKA), and C (PKC). For example, in LPS-mature dendritic cells (DCs) from a mouse model of Parkinson disease, HO-1 is regulated via AMPK (48). Also, morin (a flavonoid from fruits) down-regulates MAPK and PI3K/Akt pathways, while it induces PKA/CREB and Nrf2/HO-1 signaling in LPS-stimulated microglia (49). Besides, PKG signaling and PKC signaling show to be part of the up-regulation of HO-1 expression (50). For example, PKC α/β II is an upstream molecule of Nrf-2, required for HO-1 expression after coniferaldehyde treatment in LPS-stimulated RAW 264.7 macrophages (51). Besides, as was described above, cadmium induces HO-1 expression mediated by the PKC pathway in the BJAB cells (38). In addition, oxidized phospholipids induce HO-1 expression in human endothelial cells by the activation of PKC, PKA, and MAPK (52), and similarly, tumor necrosis factor α (TNF-α) and interleukin 1α (IL-1α) induce HO-1 expression by PKC activity (53). On the other hand, an increase in cAMP and cGMP also induces the expression of HO-1 (54). Accordingly, PKA has been studied as an upstream signal for HO-1 induction, because a large number of extracellular stimuli are capable of increasing cAMP or cGMP in the intracellular space, and this increase up-regulates HO-1 expression (55).

### Epigenetic Modulation

Gene expression can also be regulated by chromatin changes in response to environmental signals through histone modifications (56). Although the epigenetic regulation of *hmox1* is poorly studied, deacetylation and phosphorylation have been observed to be involved in the modulation of its transcription (57–59). Thus, the reduction in histone acetylation can inhibit the Mn-induced Nrf2 translocation to the nucleus and the HO-1 expression in nerve cells (57). Additionally, histone deacetylase 2 (HDAC2) has been reported to inhibit the Nfr2/HO-1 pathway in cystic fibrosis epithelial cells (58). Interestingly, the indirect involvement of histone deacetylase 6 (HDAC6) in HO-1 expression has also been reported, although the epigenetic modification on *hmox1* was not directly evaluated (60).

On the other hand, the environmental inorganic arsenite induces Nrf2/HO-1 expression in human hepatocytes (61). That occurs by the increase in serine 10 phosphorylation in histone H3 (H3S10) in the promoter region of the gene *hmox1*, activating its transcription in HaCat keratinocytes (59). Thus, although there is little information about the epigenetic modulation of HO-1, its expression also could, directly and indirectly, be regulated by epigenetic modifications.

## HO-1 Mechanisms of Immune Modulation

The HO-1 activity has been reported to impact both innate and adaptive immune responses, contributing to resolve early inflammation and limiting subsequent tissue damage (62). The function of HO-1 in the immune system is evidenced in part by the alterations reported in knockout mice. Splenomegaly, lymphadenopathy, and changes in the number of CD4^+^ T cells, as well as increased immunoglobulin M level, are observed in *hmox1*^−/−^ mice (62, 63).

Immunomodulation dependent on HO-1 activity is reported in almost all the immune cells. This broad range of action could be related both to the products obtained from heme degradation reaction and the consumption of heme *per se*, which all have protective effects (64). Heme, a complex of iron and protoporphyrin IX is the prosthetic group of heme proteins. The activity of the HO-1 is essential in the recycling of the heme group, and this is evidenced by the anemia and iron overload observed in *hmox1*^−/−^ mice (65). Under pathogenic conditions, the heme group released from the hemoproteins binds to TLR4, triggering the production of proinflammatory cytokines by macrophages (66). However, HO-1 induction in these cells not only removes heme from circulation but also triggers a functional switch toward the anti-inflammatory phenotype (67). Accordingly, HO-1 up-regulation has been extensively related to M2 polarization (68). The importance of HO-1 in cells from the myeloid linage is highlighted by conditional *hmox1*^−/−^ mice that are prone to viral infections and inflammatory conditions (69). Accordingly, CO inhibits TLR signaling pathway (70) and down-regulates the TLR4 ligand HMGB1, reducing the lethality in endotoxemia models (71). Moreover, HO-1 modulates type I interferon (IFN) production in macrophages and DCs, this effect has been suggested to be mediated by direct HO-1 binding to IFN regulatory factor 3 (IRF3) (69), as well as CO effect in IRF3 signaling (72).

On the other hand, HO-1 inhibits the LPS-induced production of inducible nitric oxide synthase (iNOS), cyclooxygenase 2 (COX2), proinflammatory cytokines, and MIP-1 in macrophages by CO and MAPK signaling (67, 68, 73). Furthermore, HO-1 induction in mast cells suppresses the degranulation and proinflammatory cytokine production (74). Interestingly, a suppressor role of HO-1 in T-cell priming of adaptive responses has been suggested. Thus, the pharmacological up-regulation of HO-1 in DCs induces a tolerogenic profile and the consequent regulatory T (Treg) cell induction (75, 76).

Pharmacological modulation of HO-1 alters CD4^+^ and CD8^+^ T-cell activity (77) and has been associated with CO and biliverdin/bilirubin reactivity (78). Regulatory T-cell activity is also modulated by HO-1, although the reported information has been more challenging to interpret. These cells show constitutive expression of HO-1, and its inhibition decreases *in vitro* Treg cell function (79); nevertheless, *hmox1*^−/−^ mice do not show Treg functionality alterations (80). Interestingly, HO-1 deficiency in DCs impacts Treg cell immunosuppressive effect (81), suggesting that HO-1 could be indirectly involved in Treg function impairment. On the other hand, biliverdin and bilirubin interfere in CD4^+^ T-cell activation (82, 83), whereas CO inhibits lymphoproliferation (78). Besides, activated Treg cells induce a suppressive phenotype in neutrophils by initiating HO-1 expression (84). Interestingly, it has been proposed that these products of HO-1 activity may remotely regulate T-cell function.

Considering that the suppressing effect of HO-1 on the immune system impacts both innate and adaptive responses, the use of inducers of this enzyme is a promising approach for autoimmune and autoinflammatory disease treatment (85). Importantly, an increasing number of reports have pointed to a regulatory effect of HO-1 and especially to its reaction end products on immune responses. In this way, the benefit of the clinical application of these products (CO, bilirubin/biliverdin) might be an exciting approach. But an intensive study of dos—effect is required because all of them possess toxic properties in higher concentrations or chronic administration (86).

## Naturally Derived HO-1 Inducers

As mentioned previously, high amounts of heme, which is the natural inducer of HO-1, have strong cytotoxic effects triggering various inflammatory events. Thus, the subsequent induction of HO-1 enzyme is considered a negative feedback mechanism that protects from the pathogenic effects of its inducer, maintaining homeostasis (87). Along these lines, importance of establishing the correct dose for each inductor has been underscored. In low doses, the heme group has been shown to have a beneficial anti-inflammatory effect depending on the activity of HO-1 and its reaction products in immune disorders (88). However, at doses that exceed the capacity of the enzyme or in individuals with other base pathogenic conditions, the heme group could have a detrimental impact. The same concept must be applied to the use of other inductors, knowing that HO-1 expression can be induced following the stimulus of several cytotoxic agents. Among them, sodium arsenite, ultraviolet A radiation, hydrogen peroxide, and structural analogs of heme have been described (7, 44, 61, 89). Special attention must be assigned to establish the correct doses of these substances to be administered (86). This is a special issue when we evaluate compounds of natural origin, which frequently are in the form of complex mixtures, not available for absorption and in very low proportions. Hence, beyond discussing the impact of a substance on immune cell function and its therapeutic application, it is critical to consider the bioavailability of the active component.

It is essential to highlight that, although HO-1 induction is efficient in many scenarios as an immunomodulatory agent, its efficiency is restricted by the availability of its substrate and the patient's health status, among others. Similar to what happens with heme, the beneficial effect of CO and bilirubin/biliverdin is observed only at low doses because they are toxic at higher concentrations. Consequently, the doses have to be adjusted, taking into account not only the possible toxicity of the inducer but also the accumulation of the enzymatic-end products.

**Figure 1:**
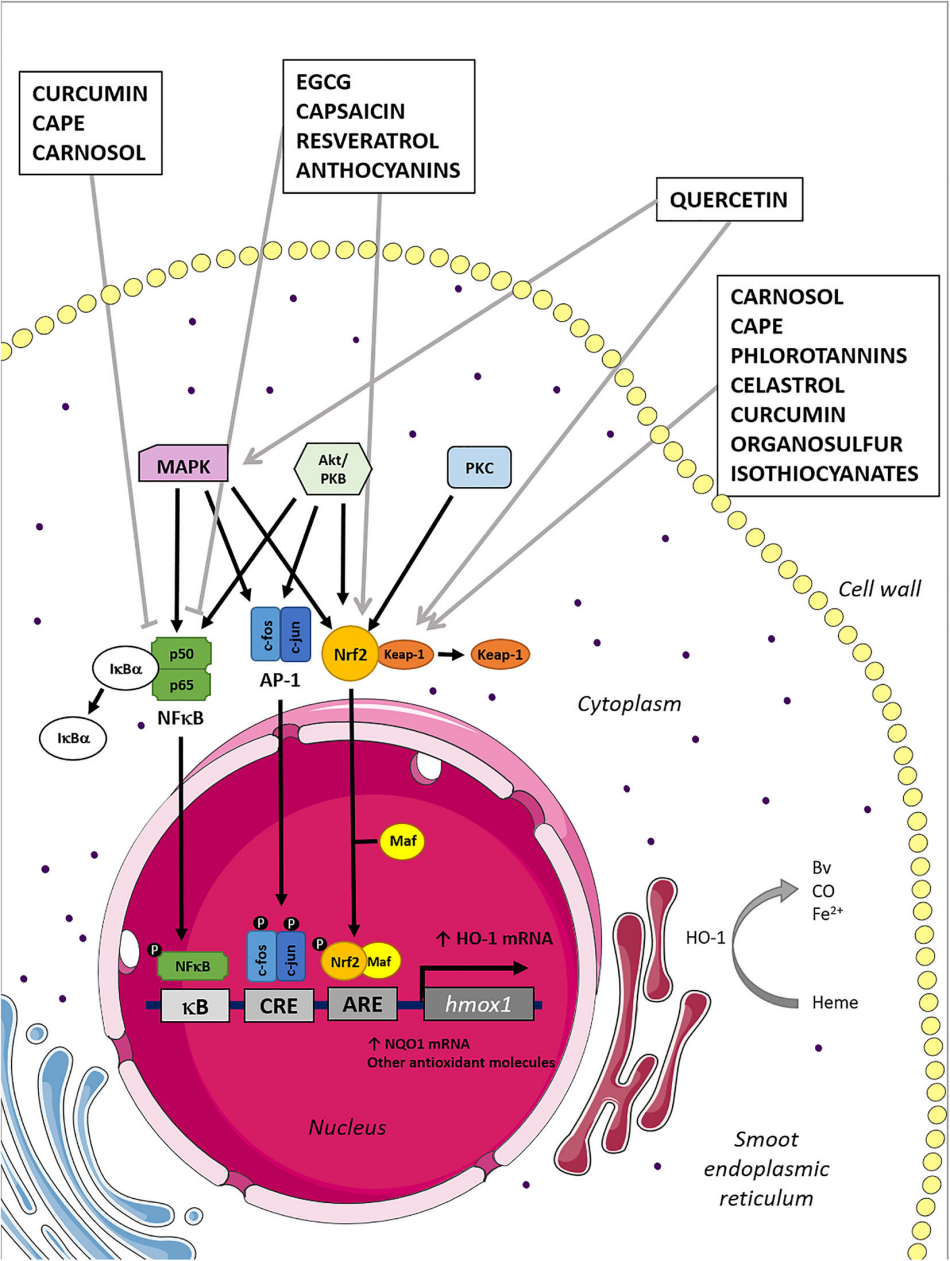
Mechanisms of HO-1 induction by naturally derived compounds. Heme oxygenase 1 induction can be mediated by several transcription factors and their upstream kinases associated, as is graphically represented in the figure. Natural compounds inhibit/activate the transcription factors, which translocate to the nucleus promoting the HO-1 mRNA expression. Consequently, the HO-1 induced expression leads to the increment of their anti-inflammatory and antioxidant products: BV, Fe^2+^, and CO.

In this context, and taking into account the detailed considerations, HO-1 inducer compounds derived from natural sources have emerged as an exciting alternative to treat inflammatory conditions. The induction mechanisms of HO-1 to the compounds discussed in this review article are schematically summarized in Figure 1. Although the bibliography describes a wide range of pharmacological properties for these molecules, given the scope of this review article, we will only focus on the effects related to the immune system by HO-1 induction.

### Quercetin

Quercetin (3,5,7,3′,4′-pentahydroxyflavone) is a flavonoid commonly found in fruits and vegetables, particularly on red onion and tea leaves (90). This phytochemical is considered a potent antioxidant synthesized by plants as a defense to environmental stress (91). Hence, it has been described as an anti-inflammatory molecule by scavenging free radicals (92). The protective antioxidant effect of quercetin has been associated with the activation of MAPK–Keap1–Nrf2–ARE signaling pathways (Figure 1) (93, 94). It has been shown that quercetin induces HO-1 at transcription and translation levels in a time- and dose-dependent manner in RAW264.7 macrophages (95) and microglia (96). Importantly, HO-1 induction in macrophages promotes the polarization toward the anti-inflammatory M2 profile (68, 97). Thus, quercetin promotes a phenotype switch in macrophages, which is beneficial in some inflammatory conditions (98, 99). Importantly, it has been reported that quercetin attenuates murine arthritis by activating HO-1 anti-inflammatory response, modulating the T_H_17/Treg balance (100), and reducing joint inflammation (101).

Importantly, *in vitro* results demonstrated the presence of quercetin-related mutagenic activity, but not seen *in vivo* (102). This difference has been attributed in part to the very low bioavailability of the quercetin. On the other hand, *in vitro* oxidation of quercetin leads to the formation of components. In contrast, the metabolism of an orally administered dose, as well as the protective mechanisms, might render the absence of carcinogenic effects *in vivo* (103). Hence, several extensive and critical reviews conclude that quercetin is unlikely to cause adverse effects in the long term.

### Curcumin

Curcumin (diferuloylmethane) is a polyphenol present in the root of *Curcuma* (*Curcuma longa*). It is a bioactive pigment responsible for the characteristic yellow color, which has been long used as a food additive and in traditional medicine as an anti-inflammatory compound (104). A large number of studies have identified curcumin as a potent inductor of the expression and activity of HO-1 in a dose- and time-dependent manner (12). It is well-known that the HO-1 expression induced by curcumin requires the activation of the Nrf2/ARE pathway (Figure 1) (12). Moreover, the inhibition of HDAC2 has been involved in HO-1 induction mediated by curcumin (58). The HO-1 up-regulation mediated by curcumin has been described to promote beneficial effects in several inflammatory pathologies (105, 106). Thus, dietary curcumin induces HO-1 mRNA and protein in DCs and impairs the differentiation of T_H_1/T_H_17 cells during experimental autoimmune encephalomyelitis (EAE) (107). Accordingly, curcumin reduces neuroinflammation in BV-2 microglial cells in an HO-1–dependent way (108). Besides, other studies demonstrated that curcumin could be useful to treat inflammatory diseases, by up-regulating HO-1 through PI3K/Akt signaling (109).

There are a large number of toxicological studies of curcumin performed in various experimental models and using different forms of curcumin (extracts, particles, suspensions, etc.). Although most *in vivo* studies do not report toxic effects (110), some *in vitro* studies showed mutagenic effects due to damage to mitochondrial and nuclear DNA using high doses (111). Although curcumin exhibits excellent anti-inflammatory properties, and researchers have published that there is little toxicity *in vivo*, their low stability, poor absorption, and rapid metabolism have promoted the development of synthetic analogs to be implemented in the clinic (112, 113).

### Carnosic Acid and Carnosol

Carnosic acid (CA) and its oxidative product carnosol are phenolic diterpenes extracted from Lamiaceae plants such as rosemary (*Rosmarinus officinalis*) and common salvia (*Salvia officinalis*) (114). Both compounds have potent anti-inflammatory and antioxidant properties (115, 116). The HO-1 up-regulation by carnosol treatment has been reported to be mediated by PI3K/Akt/Nrf2 pathway, as is shown schematically in Figure 1 (117). Besides, CA up-regulates HO-1 expression in several cell types, such as RAW264.7 macrophages (118), and suppresses the generation of ROS and nitric oxide (NO). On the other hand, it has been shown that carnosol induces HO-1 expression in DCs, reducing its production of proinflammatory cytokines and preventing the induction of T-cell responses (119). Furthermore, in another study, the DC maturation induced by LPS is reduced by carnosol through the up-regulation of HO-1, via activation of AMPK (120).

Interestingly, carnosol has also been described as one of the compounds with the best induction of HO-1/low cytotoxicity profile in BV2 microglial cells *in vitro* (121). Nevertheless, a recent study has indicated that carnosol induces DNA damage, although this activity is associated with abnormal topoisomerase activity in lymphoblastoid TK6 cells (122). Importantly, these compounds have the lowest cytotoxicity when compared to other compounds such as allyl isothiocyanate (ITC) or caffeic acid phenethyl ester (CAPE) (121). Furthermore, although diterpenes are well-absorbed orally, the bioavailability can be increased by encapsulation that protects from the degradation during digestion (123).

### Resveratrol

Resveratrol (3,5,4′-trihydroxystilbene) is a polyphenol present in many fruits and vegetables, including grapes, cocoa, peanuts, berries, and wine (124). Among the biological properties associated with resveratrol, antioxidant, anti-inflammatory, and metabolic functions have been described (125). Cytoprotective and anti-inflammatory properties of resveratrol have been related to the inhibition of nuclear factor κB (NFκB) signaling and PI3K/Akt pathway and HO-1 induction (126). Importantly, it has been reported that resveratrol induces HO-1 expression by Nrf2 activation, as schematically shown in Figure 1 (126, 127). Moreover, resveratrol induces HO-1 expression by AMPK/Nrf2/ARE pathway-dependent Jurkat cells, which in part renders the cytoprotective features of resveratrol in human T cells (128).

On the other hand, it has been observed that some resveratrol metabolites mimic some of the beneficial effects of resveratrol. Accordingly, piceatannol also induces HO-1 expression (129) and has similar cytotoxicity in RAW 264.7 macrophages (130). Despite the mentioned benefits of resveratrol, its use has been limited because of its low bioavailability (131). Thereby, several strategies have been surged as encapsulation or conjugation in nanotechnology-based carriers tending to increase their pharmacokinetics effectiveness (132).

### Anthocyanins

Anthocyanins are water-soluble pigments present in vegetables, flowers, and fruits such as berries, which confers it a bright red, blue, or purple color (133). Chemically, anthocyanins are phenolic compounds belonging to flavonoids. It is important to note that different effects have been reported using distinct anthocyanins, being cyanidin-3-glucoside (C3G), cyanidin-3-xylosylrutinoside, and cyanidin-3-rutinoside those with stronger anti-inflammatory effect (134). On the other hand, the use of individual, purified compounds exhibits a weaker effect than those observed using a mixture of both (135). On the other hand, some reports have found that antioxidant and anti-inflammatory effects of anthocyanins are associated with the Nfr2-mediated HO-1 induction (Figure 1) (136–138). Importantly, anthocyanins orally administered ameliorate inflammatory arthritis in the CIA murine model by decreasing the T_H_17 cell number and suppressing NFκB signaling (139).

Similarly, concentrated C3G-blue honeysuckle extract administration attenuates rat arthritis symptoms and enhances Nrf2/HO-1 expression and reduces iNOS and COX2 in RAW264.7 cells (140). Moreover, anthocyanins induce expression of Nrf2/HO-1 and modulate T-cell function (141). Importantly, it has been observed that C3G has proapoptotic and antiproliferative effects at concentrations found in human blood. Although a toxic *in vivo* effect remains to be demonstrated, diet consumption appears to be safe; it is essential to evaluate high doses and combined treatments (142). Importantly, anthocyanins can be absorbed all along gastrointestinal tract and metabolized by the microbiota (143). Thus, the bioavailability of anthocyanins has been suggested to be underestimated by the methods used or by not considering their metabolites (144).

### Epigallocatechin Gallate

Green tea is an infusion native from China and India, which is made from *Camellia sinensis* leaves that have not been oxidized before drying. In these leaves, the presence of between 30 and 40% of polyphenols has been estimated, and among them, epigallocatechin gallate (EGCG) or epigallocatechin-3-gallate is the most abundant catechin (145). Additionally, EGCG has been reported as the highest antioxidant activity among catechins (146). Importantly, that property is more elevated in green than black tea because of its polyphenolic content (147). The modulatory effect of EGCG on the immune system has been extensively reported, especially in T cells, where EGCG suppresses T-cell proliferation (148) and T_H_1/T_H_17 differentiation (149) but increases Treg cell differentiation (150). Although it has not been directly associated, EGCG modulation of T-cell response could be related to the up-regulation of HO-1 and Nfr2. Epigallocatechin gallate reduces renal inflammation in a cisplatin-induced nephrotoxicity model by increasing Nfr2 and HO-1 and reducing NFκB (151). However, in immune-mediated glomerulonephritis, EGCG ameliorates the inflammation without a change in renal HO-1 expression (152). Therefore, EGCG-mediated HO-1 modulation could not be involved in all pathogenic conditions. It has been shown that EGCG induces HO-1 expression and reduces transforming growth factor β (TGF-β) expression in macrophages (153). Furthermore, EGCG inhibits the production of proinflammatory cytokines and NO through HO-1 induction during adipocyte–macrophage interaction (154).

Importantly, a high dose of EGCG has been indicated as toxic to astrocytes, at least in part, by targeting mitochondria via calcium pathway (155). On the other hand, EGCG has some disadvantages, such as low stability and bioavailability, and its absorption at the intestine depends on the individual microbiota composition and its metabolism (156). All this entails a challenge for the application of this substance as a therapeutic agent, which is why several studies have developed and evaluated EGCG analogs with improved properties (157, 158).

### Phlorotannins

Phlorotannins are tannins found exclusively in marine brown algae (Ochrophyta, Phaeophyceae). Interestingly, most reports focus on phlorotannins isolated from seaweeds of Ecklonia genera (*Ecklonia cava*). Since the 70's, more than 150 phlorotannins have been extracted from several brown seaweed, many of them with anti-inflammatories properties (159). Thus, phlorotannins from *E. cava* reduce the release of proinflammatory cytokines by RAW 264.7 macrophages (160) and decrease the mortality of endotoxic shock (161). These effects have been related to the activation of the Nrf2/HO-1 pathway (Figure 1), being dieckol the phlorotannin that presents higher anti-inflammatory properties in primary macrophages (161). Furthermore, in LPS-stimulated RAW 264.7 macrophages, *E. cava* ethanolic extract treatment decreased proinflammatory cytokine gene expression and inflammatory mediators, by up-regulating Nrf2/HO-1 signaling (162). Similarly, *Ecklonia stolonifera* ethanol extract (with phlorofucofuroeckol A and B) inhibits the Akt/ERK/JNK1-2 and p38 MAPK signaling in LPS-stimulated RAW 264.7 cells with anti-inflammatory effects (163). Also, dieckol protects RAW 264.7 cells against fine dust-induced inflammation via the HO-1/Nrf2 signaling activation and inducing anti-inflammatory and antioxidant mechanisms (164). Besides, dieckol up-regulates HO-1 in LPS-stimulated macrophages, which at least in part mediates its anti-inflammatory effect (165).

The phlorotannins have not shown toxicity following oral administration to mice (166), but the growth-inhibition effect has been reported in cell lines in a dose-dependent way (166). On the other hand, phlorotannins are mainly metabolized and absorbed in the large intestine and have been reported a great interindividual variation in the metabolic profile (167). Hence, more studies are needed to evaluate the effect of food matrices and processing in phlorotannins bioavailability.

### Celastrol

Celastrol, also named tripterine, is a quinone methide triterpene used in traditional Chinese medicine, which is obtained from the root of the Thunder God Vine (*Tripterygium wilfordii*) and *Celastrus regelii* plant (168). Treatment with celastrol has been demonstrated to have beneficial effects in different forms of neurodegenerative, autoimmune, and inflammatory diseases. Celastrol induces HO-1 expression in different cell lines and has been suggested to be beneficial by reducing inflammation in some chronic diseases (169). Interestingly, celastrol inhibits proinflammatory M1 polarization in RAW264.7 macrophages via regulating Nrf2/HO-1 (170). Besides, it has been shown that synthetic derivatives of natural triterpenoids exposure on DCs result in the induction of HO-1, TGF-β, and IL-10, as well as the repression of proinflammatory cytokines (171).

On the other hand, celastrol has shown a narrow window of therapeutic *in vivo* effect, low concentrations lack efficacy, and higher levels show signs of toxicity in different models (172, 173). Besides, infertility has been indicated as an important side effect of celastrol administration (174). Thus, it has been suggested that celastrol has a dual effect, suppressing oxidative stress at low concentrations, and inducing ROS at higher levels (175). Importantly, celastrol is poorly absorbed after oral administration in rats, and it is absorbed more efficiently by female rats as compared to males. However, bioavailability can be increased by Thunder God Vine extract administration (176), suggesting additional components in the extract aid to celastrol absorption. Hence, new celastrol analogs have been developed with higher pharmacokinetics properties and lower toxicological characteristics (175). In addition, several celastrol derivatives have been synthesized to improve its bioavailability for therapeutic administration (177).

### Caffeic Acid Phenethyl Ester

Caffeic acid phenethyl ester is the ester of caffeic acid extracted from honeybee propolis, which has been used for many years in traditional medicine (178). This compound has been characterized by its strong antioxidant and cytoprotective properties, as well as immunomodulatory and anti-inflammatory attributes (179). It has been described that CAPE inhibits cytokine production by stimulated DCs (180) and suppresses DNA synthesis of human peripheral blood mononuclear cells (PBMCs) in response to mitogens (181). Besides, CAPE has been identified as a potent HO-1 activator (12), which induces Nrf2 and in turn inhibits NFκB activation in macrophages (Figure 1) (182, 183). Caffeic acid phenethyl ester derivative compounds promote the switch of macrophage phenotypes from proinflammatory M1 to resolving M2. Besides, the effect is dependent on the activation of the Nrf2/HO-1 pathway (184). Moreover, CAPE induces HO-1 in microglia cells, reducing NO production (185).

Interestingly, new CAPE analogs have been developed that show a more potent HO-1 induction (186). In fact, it has been indicated that HO-1 up-regulation plays an essential role in the cytoprotective activity of CAPE derivatives than their antioxidant activity (187). Besides, its bioavailability has been shown to increase after glycosylation without affecting the CAPE anti-inflammatory properties (188). Propolis has a low order of acute oral toxicity (189), and importantly, no significant clinical toxicity has been reported in animals after oral propolis extract administration (190).

### Capsaicin

Capsaicin (*trans*-8-methyl-*N*-vanillyl-6-nonenamide) is the active ingredient of chili peppers, which is found in the placental tissue that surrounds the seeds in *Capsicum* spp (191). This pungent oleoresin has shown strong anti-inflammatory properties (192, 193). The interaction of capsaicin with transient receptor potential vanilloid 1 is responsible for nociceptive, thermal, and mechanical sensations and has been shown to induce HO-1 expression (194). This receptor is present in almost all tissues, including the immune system. Moreover, it has been reported that capsaicin induces the expression of antioxidant enzymes by phosphorylation of Akt, modification of Keap1 protein, release, and translocation of Nrf2 to the nucleus and by binding to ARE elements to induce HO-1 expression (Figure 1) (195). Furthermore, it has been reported that capsaicin has therapeutic potential in renal damage by attenuation of the expression of inflammatory mediators (196). However, this effect is entirely abrogated by the treatment with the HO inhibitor ZnPP (197). Moreover, capsaicin increases HO-1 expression and inhibits NO production in LPS-stimulated RAW264.7 macrophages (198).

Interestingly, although capsaicin is highly absorbed, its half-life in plasma is low, and therefore, novel drug delivery strategies have been evaluated to improve bioavailability, such as the use of capsaicin-loaded polymeric micelles (199). Importantly, capsaicin has shown both mutagenic and carcinogenic activities, but results are conflicting. Thus, other studies indicate that capsaicin possesses chemoprotective activity against some carcinogens and mutagens chemical (200). Therefore, toxicity is determined only in animals with high median lethal dose (LD50) values, and there are no reported cases of an overdose in humans (201).

### Garlic-Derived Organosulfur Compounds

Organosulfur are bioactive components of garlic (*Allium sativum*) essential oil, mustard, asafoetida, and other food extracts (202). Worldwide, the traditional use of garlic in medicine is known for thousands of years, and multiple pharmacological properties have been reported in the literature and applied to clinical trials (203). The presence and abundance of compounds in garlic vary according to preparation and extraction (204), suggesting that there is also a wide variety of immunoregulatory properties (205). Among organosulfur present in garlic, diallyl sulfide, diallyl disulfide (DADS), and diallyl trisulfide (DATS) are the major inducers of HO-1 expression in human hepatoma HepG2 cells (206). It has been reported that DADS induces Nrf-2/ HO-1 signaling (Figure 1) and reduces proinflammatory cytokine production in LPS-stimulated RAW 264.7 macrophages (207). Moreover, S-allyl cysteine (SAC), an organosulfur present in aged garlic extract, has antidiabetic, antioxidant, anti-inflammatory properties, and helps in preserving cognitive deficits in diabetic rats through the regulation of Nrf2/NF-kB/TLR4/HO-1 signaling cascade (208). Furthermore, aged red garlic extract reduces LPS-induced NO production in RAW 264.7 macrophages, and this effect is dependent on HO-1 induction (209).

Despite toxicological data of organosulfur from garlic are limited; *in vitro* studies report no mutagenicity (210). Besides, oral administration of allium extracts showed no mortality or side effects in rats (211). Importantly, the beneficial effect of these compounds is closely related to its bioavailability, and therefore thermal instability of these compounds must be considered (212).

### Isothiocyanates

Naturally occurring ITCs can be found in cruciferous vegetables (213). They have been evaluated as immunoregulatory and antioxidant molecules in many reports (214–216). Sulforaphane, a naturally occurring ITC from broccoli, has been demonstrated to attenuate cell damage induced by 1-methyl-4-phenyl pyridine ion (MPP+) in PC12 cells by reversing the reduced expression of Nrf2, HO-1, and NAD(P)H-quinone oxidoreductase (NQO1) (217). The beneficial effect of SFN-induced Nrf2-HO-1/NQO-1 signaling pathway activation was also demonstrated in chronic renal allograft dysfunction (Figure 1) (218). Furthermore, SFN suppresses T_H_17 response on untransformed human T cells by decreasing GSH and the accumulation of ROS (219). Interestingly, SFN also inhibits the inflammatory response by suppressing the cytokines response, NFκB activation, and inducing HO-1 expression in cultured monocytes and the lungs of mice (220). Besides, SFN prevents the production of NO and cytokines by activating the Nrf2/HO-1 signal transduction pathway and limiting iNOS activity (221). Furthermore, SFN reduces IL-23 and IL-12 production in DCs (222).

Finally, the bioavailability of ITCs has been evaluated in broccoli sprouts and has been reported that it is highly influenced by the food structure and composition (223), without metabolites accumulation (224). Importantly, although SFN is considered safe at low doses (225), it has been reported that high doses have proconvulsant effects and produce marked sedation, hypothermia, impairment of motor coordination, and deaths (226). Despite that doses are higher than found in dietary consumption, the risk–benefit ratio of SFN administration must be considered when the diet is supplemented.

## HO-1, Natural Compounds, and Immune-Mediated Diseases

**Figure 2:**
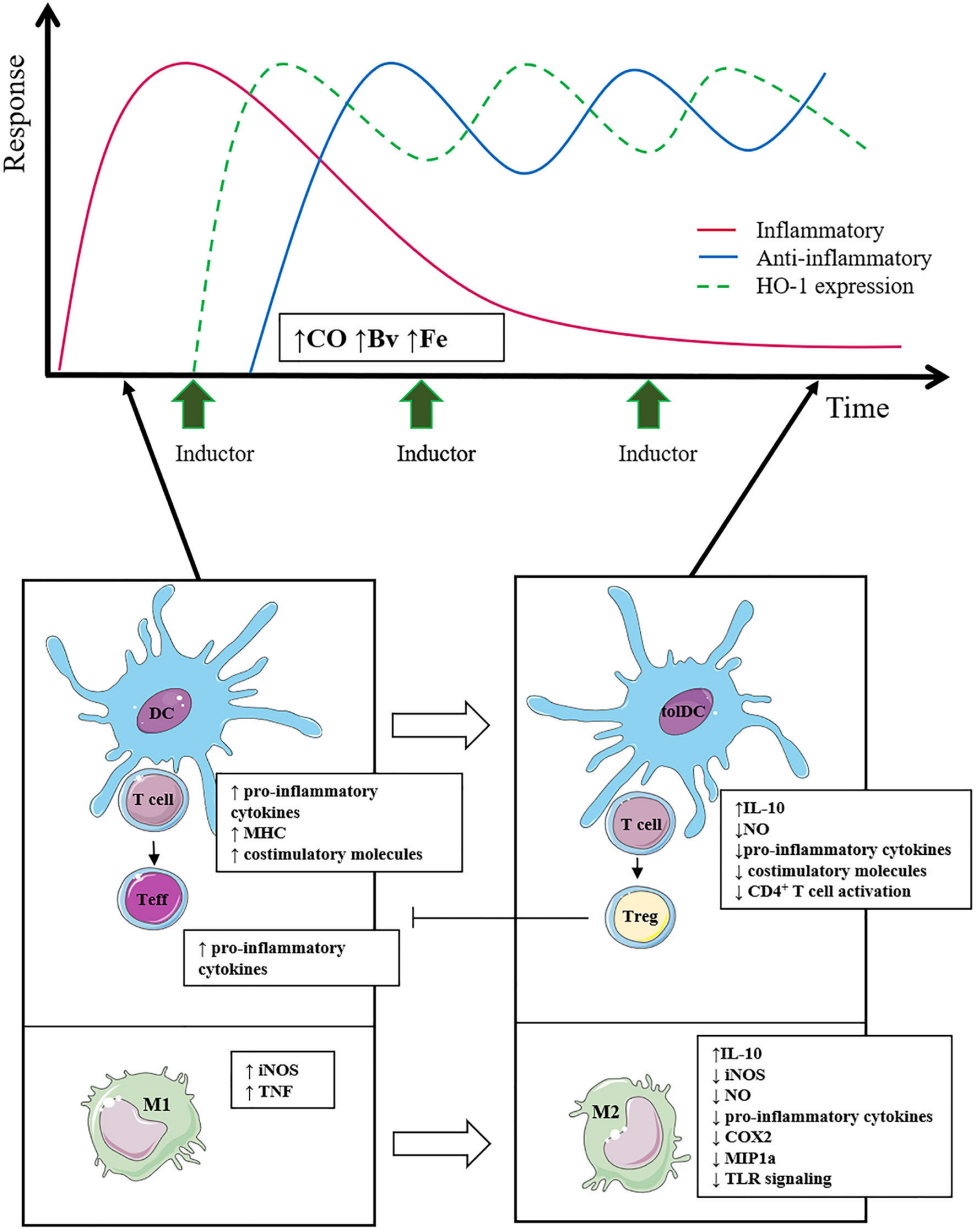
The effect of the use of HO-1 inducers on the inflammatory and anti-inflammatory response is outlined. Multiple inducer pulses keep HO-1 expression elevated over time (green line), whereas the anti-inflammatory response (blue line) increases accordingly. Consequently, the inflammatory response (red line) is reduced in a sustained way, managing to limit tissue damage. The induction of HO-1 in immune cells promotes the polarization of macrophages toward the anti-inflammatory M2 profile and in turn favors a tolerogenic profile in DCs, which together reduce T-cell activation and promote Treg cell differentiation.

The beneficial role of HO-1 induction in autoimmune and inflammatory disorders has been extensively reported in the last decades (227). In fact, HO-1 knockout mice develop a chronic inflammatory disease with increased peripheral blood lymphocyte count and accumulation of polymorphonuclear cells and monocytes/macrophages in the spleen (65). Consistently, a polymorphism in the *hmox1* promoter region, which regulates the HO-1 induction (228), has been associated with increased systemic lupus erythematosus (SLE) (229) and rheumatoid arthritis (RA) susceptibility (230). On the other hand, as most of the modulatory effects of HO-1 are described on innate cells, it could be assumed that these inductors would be more efficient for the treatment of autoinflammatory diseases, especially with myeloid cell expansion. Nevertheless, HO-1 induction has also been beneficial for the treatment of autoimmune diseases mediated by T or B cells (10). In this sense, it is essential to highlight that, in the first place, the conditions described below are immersed in a scenario that involves characteristics of both autoimmune and autoinflammatory disorders (85). Second, the role of DCs as an interconnection between innate and adaptive responses is critical. Hence, as mentioned previously (Figure 2), the induction of HO-1 in DCs has been described to induce a tolerance profile in DCs (76), which in turn is capable of suppressing subsequent autoreactive responses. Therefore, the induction of HO-1 expression has been proposed as a strategy to improve autoimmune diseases (10). For this purpose, some substances of natural origin capable of triggering the overexpression of HO-1 in animal models have been evaluated in recent decades (Table 1).

**Table 1:** Natural source, evaluated model of autoimmune diseases, and reported effect of natural compounds.

**Reported compound**	**Natural source**	**Evaluated model**	**Reported effect**	**References**
Quercetin	Fruits and vegetables (particularly red onion and leaves tea)	CIA mice	↑HO-1 protein in synovium, modulation of Th17/Treg balance, ↓pro-inflammatory cytoquines	(100)
		DSS-induced colitis mice	↑HO-1 protein in intestinal macrophages, ↑IL-10, ↓TNF-α, IFN-γ, IL17A, and IL-6, ↓intestinal histological score	(231)
Curcumin	*Curcuma Longa*	STZ-induced T1D rats	↑HO-1 mRNA in liver, ↓blood glucose	(105)
		STZ-induced T1D rats	↑HO-1 mRNA and activity in pancreas, aorta, and liver, ↓blood glucose, ↑plasma insulin	(232)
		Nephrectomized rats IBD	↑HO-1 protein in kidney, ↓TGF-β, TNF-α, and COX2	(233)
Resveratrol	Fruits and vegetables	STZ-induced T1D mice	↑HO-1 mRNA and protein in testicular tissues	(234)
		STZ-induced T1D rats	↑HO-1 protein, ↓TGF-β in heart	(235)
		Osteoarthritis-induced rats	↑HO-1 protein in joint tissue, ↓NF-kB protein, ↓TNF-α, IL-1β, IL-6, and IL-18	(236)
Epigallocatechin gallate (EGCG)	Green tea	DNBS-induced colitis rats	↑HO-1 protein in colon, ↓MPO, ICAM-1, and TNF-α ↓diarrhea	(237)
Caffeic acid phenethyl ester (CAPE)	Honeybee propolis	STZ-induced T1D rats	↑HO-1 protein, ↓blood glucose, ↑plasmatic insulin, ↓iNOS,	(238)
Garlic-derived organosulfur compounds (DAS, DADS, DATS, SAC, AMS)	*Allium sativum*, mustard, *Ferula assafoetida*	STZ-induced T1D rats	↑HO-1 mRNA and protein in heart, ↓cardiac injury	(239)
Isothiocyanates (sulforaphane, phenethyl isothiocyanate, allyl isothiocyanate)	Cruciferous vegetables	DSS-induced colitis mice	↑HO-1 mRNA in colon, ↓clinical score, ↓TNF-α, NO, MPO, IL-1, IL-6, and iNOS	(240)

*Only studies where the HO-1 induction was evaluated are listed*.

### Multiple Sclerosis

**Figure 3:**
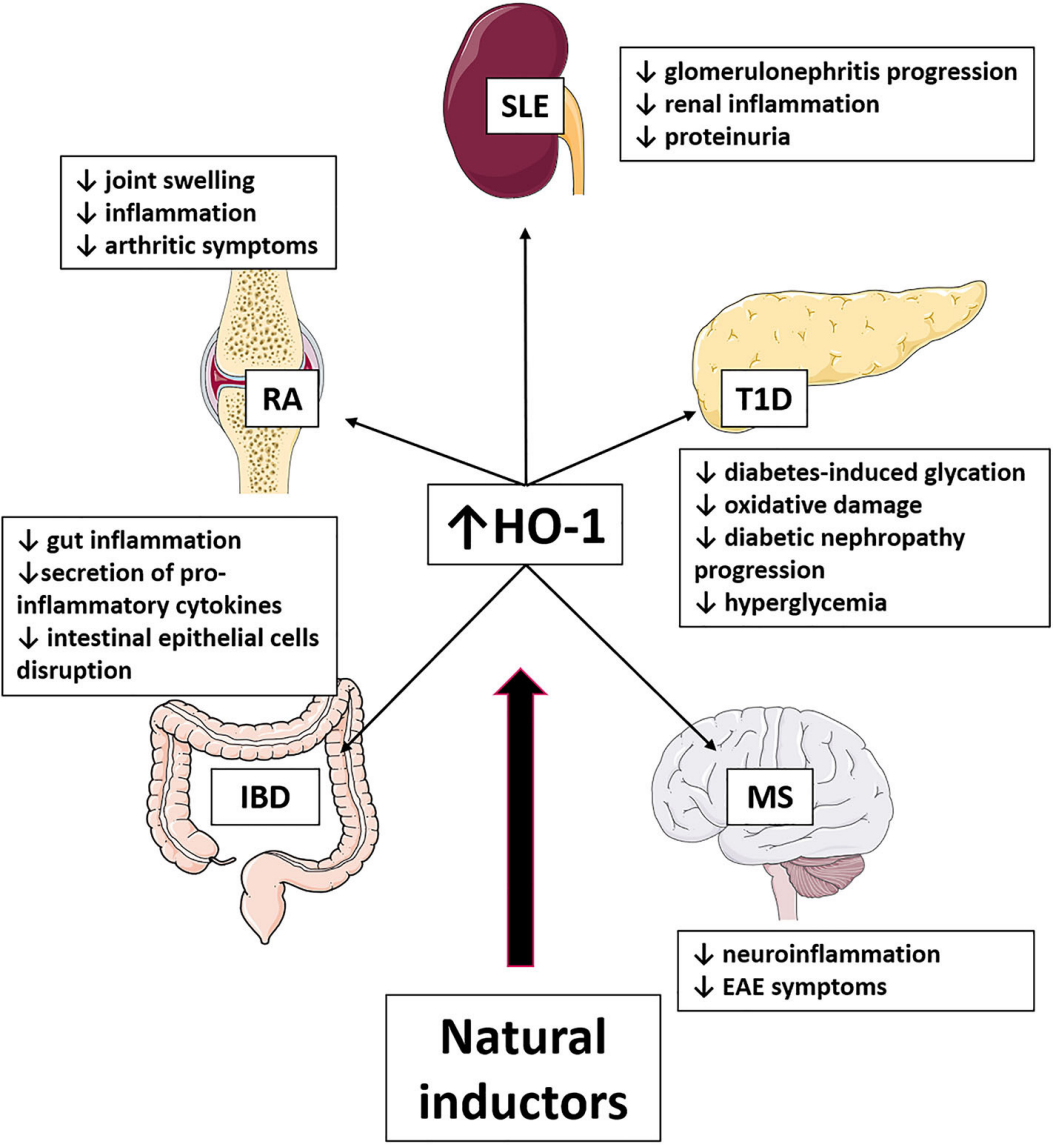
Effect of HO-1 induction produced by natural compounds in autoimmune diseases. The use of natural inductors of HO-1 has been shown in SLE, T1D, EAE, IBD, and AR to produce the beneficial effects listed in the figure.

Multiple sclerosis (MS) is a demyelinating autoimmune pathology that affects the central nervous system (CNS) in humans (Figure 3) (241). Interestingly, it has been observed that HO-1 expression is reduced in PBMCs from MS patients during exacerbation periods (242). Accordingly, CO treatment has been suggested by several *in vivo* preclinical studies as an effective treatment for MS (243). Dimethylfumarate (DMF), a small molecule that improves psoriasis and MS, has been reported to induce a tolerogenic profile in DCs by HO-1 expression (244). Significant contributions aiming to understand the role of inflammation and the immune system in MS have been made using an animal model named EAE (245). Heme oxygenase 1 deficiency in mice that suffer EAE has been shown to develop an aggravated disease (246). Moreover, the protective effect of HO-1 induction has been associated with inhibition of MHC II expression by antigen-presenting cells, including DCs, microglia, and infiltrating macrophages. Importantly, inhibition of CD4^+^ and CD8^+^ T cell accumulation and effector function in CNS have also been reported (246). Besides, intraperitoneal CAPE administration inhibits ROS production in EAE and ameliorated clinical symptoms in rats (247). In addition, CAPE treatment possesses antineuroinflammatory effects, which are related, at least in part, to the increased expressions of HO-1 via AMPKα in microglial cells (185). Besides, CAPE inhibits the expressions of iNOS, COX2, and NO production in microglia, showing an antineuroinflammatory effect (185). Importantly, quercetin mitigates inflammatory responses in microglial cells inhibiting LPS-induced NO production by HO-1 induction (96). Similarly, curcumin reduces neuroinflammation by HO-1 induction in BV-2 microglial cells and reduces iNOS and COX2 expression (108). Despite, these natural compounds have anti-inflammatory effects mediated by HO-1 *in vitro* in microglia, the HO-1 contribution in the improvement associated with natural compounds has not been evaluated in models *in vivo*. Thus, several other natural compounds have been reported to improve MS symptoms, although a direct involvement of HO-1 induction has not been elucidated.

### Type 1 Diabetes

Type 1 diabetes (T1D) is a chronic autoimmune disease characterized by the presence of islet autoantibodies and autoreactive T cells, pancreatic beta cell loss, and hyperglycemia (Figure 3) (248). Up-regulation of HO-1 in DCs prevented the increase in glycemia in non-obese diabetic (NOD) mice and a lower T1D incidence, suggesting it as a potential therapeutic approach for T1D treatment (249). Furthermore, a short-term induction of HO-1 promotes the recruitment of mesenchymal cells, M2 macrophages, and fibrocytes with repair properties, preventing T1D development in rats (250). Interestingly, genetic restoration of HO-1 expression in DCs from NOD mice reduces T1D incidence highlighting their role in tolerizing autoreactive T-cells (249). It has been shown that resveratrol administration in drinking water attenuates biochemical changes associated with diabetes, and this protective role is mediated by TGF-β reduction and HO-1 induction (235).

Additionally, resveratrol administered by gavage attenuates T1D-induced testicular oxidative stress and apoptosis by Akt-mediated Nrf2 activation and Keap1 degradation (234). On the other hand, curcumin administration in STZ-induced T1D decreases the blood glucose concentration via the activation of the Keap1–Nrf2–AREHO-1 signaling pathway, although no immunological mechanisms were described in the study (105). In the same model, a small dose of water-soluble curcumin derivative orally administered has been shown to have antidiabetic properties mediated by HO-1 induction (232). These reports suggest that curcumin administration reduces oxidative stress in part by HO-1 induction ameliorating symptoms in T1D models. Finally, it has been proposed that appropriate DATS consumption might be a cotherapy for hyperglycemia (251). Besides, DATS perioperative intraperitoneal administration to STZ-induced T1D rats reduces injury at least in part by up-regulating the Nrf-2/HO-1 antioxidant signaling and reducing myocardial apoptosis (239). Accordingly, it has been reported that the impairment of Nrf-2/HO-1 signaling contributes to aggravated myocardial injury in STZ-induced T1D mice (252). Moreover, antihyperglycemic property of CAPE used in the STZ-induced T1D rat model has been associated to HO-1 induction (238). It is important to highlight that although various HO-1–inducing compounds have been evaluated in the context of T1D models with promising results, more studies of their effect on the immune response are necessary.

### Rheumatoid Arthritis

Rheumatoid arthritis is a chronic autoimmune disease characterized by articular cartilage erosion and inflammatory cell infiltration in the joints, leading to disability (Figure 3) (253). It has been observed that HO-1 is highly increased in synovial fluid (254) and peripheral monocytes (255) from RA patients, suggesting that HO-1 expression might be an inflammation marker. In addition, HO-1 is up-regulated in the murine collagen-induced arthritis (CIA) (256) and the rat adjuvant-induced arthritis model (257). Interestingly, high levels of HO-1 could be, at least in part, an adaptive mechanism for limiting inflammation and cytotoxicity (256).

The therapeutic effect of quercetin administration by gavage has been evaluated in the CIA model showing anti-inflammatory results. Importantly, quercetin oral administration reduces arthritic manifestations in the CIA murine model, by the decrease in proinflammatory cytokines (TNF-α, IL-1β, IL-6, IL-17A, IL-21) and increase in IL-10 and TGF-β and the restoration of T_H_17/Treg balance. Remarkably, HO-1 siRNA inhibits the beneficial effect of quercetin, suggesting a critical role of HO-1–mediated anti-inflammatory response (100). Moreover, oral resveratrol administration decreases inflammatory damage of autoinflammatory osteoarthritis in rats via Nrf-2/HO-1 expression in joint tissue by reducing TNF-α, IL-1β, IL-6, and IL-18 expression (236).

Although beneficial effects have been reported in RA by administering natural compounds such as anthocyanin, celastrol, or garlic derived organosulfur, there is no direct association with HO-1 induction.

### Systemic Lupus Erythematosus

The SLE is a chronic autoimmune disease that could affect several organs such as kidneys, joints, skin, nervous systems, and mucosa, among others (Figure 3) (258). This disorder is characterized by the production of autoantibodies against nuclear self-antigens and immune complex deposition, which are associated with organ malfunction and injury (259). Based on the inhibitory effects of tolerogenic DCs (tolDCs) in T-cell priming and B-cell differentiation and the impact of these cells in maintaining peripheral tolerance (260), tolDCs administration has been suggested as a therapy in the progression of SLE (9, 261). Accordingly, the transference of tolDC generated with HO-1 inductor cobalt (III) protoporphyrin IX (CoPP), dexamethasone, and rosiglitazone improves SLE symptoms in mice, such as decreased antinuclear antibodies, skin lesions severity, and clinical score (262).

Moreover, HO-1 induction confers an anti-inflammatory profile to monocytes and DCs, and accordingly, is less expressed in monocytes from SLE patients, suggesting that HO-1 deregulation may be involved in the development or progression of SLE (263). Therefore, CO exposure reduces the clinical score by a decrease in the expansion of CD11b^+^ cells (261) and leukocyte activation in SLE mice (6). Consequently, it has been proposed that the application of HO-1 inducers could be an appropriate therapy to ameliorate SLE conditions. For example, a diet with extra virgin olive oil was shown to improve renal damage in an SLE model in mice through the induction of Nrf-2/HO-1 expression and reduction of proinflammatory cytokines by splenocytes (264). Besides, oral curcumin administration reduces renal fibrosis and inflammation mediated by Nrf2 and HO-1 induction in nephrectomized rats (233). However, further studies are needed to elucidate the precise role of HO-1 in quercetin, resveratrol, and celastrol–lupus amelioration.

### Inflammatory Bowel Disease

Among chronic inflammatory bowel diseases (IBDs) are Crohn disease (CD) and ulcerative colitis (UC), which are characterized by symptoms of diarrhea, abdominal pain, and the presence of blood in the stool (Figure 3). Both UC and CD are considered polygenic autoinflammatory diseases (85). Although CD involves inflammation at any gastrointestinal section, UC is restricted to colonic inflammation. Recently, several studies have suggested that HO-1 and its products could have an critical role in the modulation and progression of IBD (11). The pharmacological induction of HO-1 has been extensively reported to reduce gut inflammation by anti-inflammatory cytokines (11). Thus, in the model of UC triggered by the administration of dextran sulfate sodium (DSS), the induction of HO-1 by CoPP or hydrogen-rich water reduces the intestinal histological damage (265, 266). Thus, a significant reduction in TNF-α, IL-6, and IL-1β proinflammatory cytokines has been reported (266). Besides, some drugs such as tranilast, *Atractylodes macrocephala*, or *Taraxacum* herb extracts, which ameliorate symptoms in IBD patients and exert their effect by inducing HO-1 expression (11).

In a recent study, it was observed that oral quercetin ameliorates T-cell–mediated UC, reduces gut inflammation, and modulates intestinal macrophages inducing an anti-inflammatory M2 profile and inhibiting CD4^+^ T cell activation (231). Importantly, macrophage depletion partially blockades the beneficial effect of quercetin in gut inflammation, highlighting the role of these cells in intestinal homeostasis (231). Moreover, curcumin protects human intestinal epithelial cell disruption and barrier dysfunction via HO-1 induction (267). Also, green tea administration up-regulates HO-1 expression in the colon, which may contribute to the protective effects in 2,4,6-dinitrobenzenesulphonic acid–induced colitis model by reduction of colonic myeloperoxidase (MPO) and TNF-α production (237). Interestingly, it has been reported that both resveratrol and C3G induce HO-1 in HT-29 intestinal cells, which may interfere with the expression of proinflammatory enzymes (268). Thus, HO-1 induction has been suggested as a putative molecular mechanism associated, at least in part, to the therapeutic effect of resveratrol. On the other hand, the gavage administration of ITCs in the DSS-induced colitis model ameliorates the severity of the disease, mediated by Nrf2 and HO-1 anti-inflammatory/antioxidant signaling pathway (240). Importantly, ITC administration decreases colonic secretions of proinflammatory TNF-α, NO, and MPO in UC, besides reducing gene expression of IL-1, IL-6, TNF-α, and iNOS (240). Hence, IBD characterized by self-directed inflammation, where the activation of innate immune cells plays a critical role in pathogenesis, appears to be a particularly promising target for the implementation of HO-1 inducers as immunomodulators.

## Concluding Remarks

Heme oxygenase 1 induction has been suggested as a therapeutic approach to ameliorate self-directed immune diseases, including both autoimmune and autoinflammatory diseases. Accordingly, many dietary and herbal medicines that induce HO-1 expression have been widely evaluated as a possible strategy to improve autoimmunity. Thus, consumption of spicy food, tea, or red wine might modulate immune responses. However, it is crucial to consider the bioavailability, absorption, toxicity, and metabolism of these compounds, as well as reported discrepancy among cell culture assays and *in vivo* results. Furthermore, the whole picture during a classical immune response, a self-reactive response, or an exacerbated inflammation continues to be a significant debt in HO-1 scientific research.

It is important to note that most HO-1–inducing compounds have been associated with a certain degree of toxicity, especially in studies *in vitro*. In the case of the heme group, a nocive event leads to reduce excessive inflammation and maintain homeostasis. Thus, and taking into account also the bivalent immunosuppressant/toxic nature of the HO-1 reaction products, a more exhaustive study of the inducer doses *in vivo* is necessary.

Experimental data evidencing worthy properties of bioactive substances (from plants and other natural sources), which induce HO-1 expression is continuously increasing. Nevertheless, a critical evaluation of the literature data is essential, first because the majority of studies are conducted on *in vitro* models, and thus, it is crucial to test natural HO-1 inducers in different *in vivo* models, and second, because most studies highlight their experimental results underestimating the detailed report in chemical obtention methods for single molecules from food extracts, impaired reproducibility, and delay in its wide prescription.

## Author Contributions

SF, MR, AF-F, and CC wrote the manuscript. CR, SB, JM-O, and AK proofread the manuscript and corrected language use. SF constructed the figures. AK supervised the work and performed critical revision of the manuscript. All authors revised and approved the manuscript.

## Conflict of Interest

The authors declare that the research was conducted in the absence of any commercial or financial relationships that could be construed as a potential conflict of interest.

## Abbreviations

HO-1: Heme oxygenase 1
CO: Carbon monoxide
StRE: Stress-responsive elements
DCs: Dendritic cells
NFκB: Nuclear factor κB
NF-E2: Nuclear factor–erythroid 2
Nrf2: NF-E2-related factor 2
AP-1/2: Activador protein 1/2 families
ARE: Antioxidant response element
Keap1: Kelch-like ECH-associated protein 1
MAPKs: Mitogen-activated protein kinases
PKA: Protein kinase A
PKC: Protein kinase C
PI3K: Phosphatidylinositol 3-kinase
EGCG: Epigallocatechin gallate
ROS: Reactive oxygen species
SLE: Systemic lupus erythematosus
RA: Rheumatoid arthritis
CAPE: Caffeic acid phenethyl ester
DADS: Diallyl disulfide
DATS: Diallyl trisulfide
NQO1: NAD(P)H-quinone oxidoreductase
CIA: Collagen-induced arthritis
ITC: Isothiocyanate
SFN: Sulforaphane
ERK: Extracellular regulated kinases
JNK: c-Jun N-terminal kinases
HDAC2: Histone deacetylase-2
CA: Carnosic acid
T1D: Type 1 diabetes
NOD: Non-obese diabetic
STZ: Streptozotocin
CoPP: Cobalt (III) protoporphyrin IX
IBD: Inflammatory bowel disease
CD: Crohn's disease
UC: Ulcerative colitis
DSS: Dextran sulfate sodium
MARE: Maf recognition elements
LPS: Lipopolysaccharide
cAMP: Cyclic adenosine monophosphate
cGMP: Cyclic guanidine monophosphate
IRF-3: Interferon regulatory factor-3
C3G: Cyanidin-3-glucoside
ZnPP: Zinc protoporphyrin
MPP+: 1-methyl-4-phenyl pyridine ion
EAE: Experimental autoimmune encephalomyelitis
CNS: Central nervous system
MS: Multiple sclerosis
DMF: Dimethylfumarate
tolDC: Tolerogenic dendritic cell.
